# The Research of the Rolling Speed Influence on the Mechanism of Strip Breaks in the Steel Rolling Process

**DOI:** 10.3390/ma13163509

**Published:** 2020-08-09

**Authors:** Ján Rusnák, Peter Malega, Jozef Svetlík, Vladimír Rudy, Norbert Šmajda

**Affiliations:** 1 Cold Rolling Mill Quality, U. S. Steel Košice, s.r.o, Vstupný areál U. S. Steel, 044 54 Košice, Slovakia; janrusnak2@sk.uss.com; 2 Institute of Management, Industrial and Digital Engineering, Faculty of Mechanical Engineering, The Technical University of Košice, Letná 9, 040 01 Košice, Slovakia; peter.malega@tuke.sk (P.M.); vladimir.rudy@tuke.sk (V.R.); 3 Department of Manufacturing Machinery, Faculty of Mechanical Engineering, The Technical University of Košice, Letná 9, 040 01 Košice, Slovakia; 4 SENZOR® s.r.o. Košice, Park Angelinum 19, 040 01 Košice, Slovakia; norbertsmajda@gmail.com

**Keywords:** strip breaks, foreign materials, defects, rolling forces

## Abstract

This paper evaluates the results of research aimed at changing the rolling speed and the effect of foreign particles in the steel strip, as well as the forces in the rolling process. It also compares the correlation of lab results, theoretical expectations and real-life observations. It supplements the already existing practices aimed at strip-break elimination that were developed and implemented worldwide. Records from a five-stand tandem mill were used for the data analysis. The historical databases developed based on incidents (strip breaks) since 2013 were used; the detailed position of each strip break was documented, along with defects found at the portions of steel strips that broke or the information that no defect was found. The paper contains an evaluation of metallographic analyses of the samples of the strip breaks.

## 1. Introduction

Steel producers face many challenges in the steelmaking process, and amongst the most serious are strip breaks in the rolling process [1,2]. A strip break during rolling often damages the line, work rolls and back-up rolls. It also causes delays and product damage [3,4]. We must take into consideration all the processes the semi-product goes through from the Blast Furnace operation to the cold-rolling mill, on top of the significant economic losses. Such event also has a negative impact on the environment, as the strips that do not pass through the production flow need to be reproduced to satisfy the customer’s order [5].

The result of the research of the case study made by a TATA Steel India steelmaking team was the elimination of slag in slab-casting process [6]. If any slag and/or other non-metallic inclusions are present in steel, they recommend rolling the slabs to heavier gauges to reduce the risk of strip breaks.

The team, led by Zheyuan Chen [7,8] worked on the elimination of strip breaks during rolling through advanced data analysis. They managed to establish twenty key parameters that influence strip breaks, but they did not succeed in determining an explicit root cause of strip breaks. Their conclusion was the decision to carry out the research, focusing on the data that provide more accurate details with data about the process.

The other research paper of the team led by Zheyuan Chen [7,8] on the elimination of strip breaks in the rolling process proposes the implementation of a width-meter at both the entry and exit end of the rolling mill. This theory is based on strip necking during rolling. In the rolling process, the strip necking coefficient is set-up; if the strip is too tight, its width narrows, therefore it strays from the predicted dimensions—this means that the strip is very close to breaking. If the threshold values are achieved, the strip-shape mechanism is triggered, reducing the tension—this should prevent strip breaks. 

The research team from Yanshan University in China led by Xiao Liu [9] engaged in the research of this study, providing theoretical guidance for practical production by defining the product specification ranges and rolling regulations for existing rolling mills and determining the roller diameters, force, rolling speed and energy parameters for the design of rolling mills. 

The team led by Bhattacharya D [10] carried out research on the root cause of strip breaks in the cold-rolling process, which are defects arising in the process of casting slabs. Defects that arise in the casting process are brought to the surface in the rolling process by gradually reducing the thickness of the material, making them critical in terms of tearing the strip in the rolling process. The conclusion of the work of the Bhattacharya D team [10] confirmed the results that are elaborated on in this paper.

A trio of authors, S.B. Chaudhury, J. Bhattacharya and B. Puthal [11], provided research into the field of strip-break tearing in the cold-rolling process. The result of their work is detection logic, based on the winding force, speed of the stands and tensioning of the stands. It detects strip breakage during the rolling process. This article provides a solution and view of the problem in terms of reducing the consequences of tearing the strip in the rolling process.

The authors V.L. Mazur and O. V. Nogovitsyn [12], in the Theory and Technology of Sheet Rolling: Numerical Analysis and Applications, investigate the conditions of stability and reliability of the hot-rolling process which forms the input substrate of the cold rolling which decisively influences the possible tearing of the strip in the cold-rolling process. The publication further examines the conditions of friction, deformation and stability of the cold-rolling process.

Three researchers, Michel Gérard, Mike Hastings, and Philippe Vervoort [13], at Arcelor Steel Belgium Liège, carried out research to eliminate resonance in the cold-rolling process. The uncontrolled tandem resonance in the rolling process results in the tearing of the strip. As stated in the article in the research process, they dealt with influences that enter the rolling process, evaluating the imperfections of the back rolls, working rolls and physical conditions in the rolling process.

The team led by Kwon H.C. [14] carried out research about the surface wrinkle defect of carbon steel in the hot bar rolling process. According to the simulation results of this study, temperature and specific deformation energy levels were the most important parameters to govern the formation of surface defect. Finally, it was found that the specific deformation energy level was dependent on the roll geometry. Thus, by modifying the roll geometry, the instability can be reduced according to the limiting value of the specific deformation energy.

Research conducted by Li et al. [15] deals with the fact that a slab’s internal defects that are associated with porosities significantly influence the qualities of hot-rolled plates, especially those which are rolled with a low reduction ratio. Hot-core heavy reduction rolling (HHR2) is an innovative technology to eliminate the porosity defects in casting steel through a single pass rolling, after the final solidification point. The experimental results showed that both the microstructure homogeneity and the uniformity of the mechanical properties along the thickness of the plate manufactured using the HHR2 process were clearly improved. In addition, the microstructure-inheritance mechanisms of the HHR2 process at three different process stages: continuous casting, reheating, and multi-pass hot rolling, were discussed.

The authors Teschke, M., Rozo Vasquez, J., Lücker, L. and Walther, F. [16] found that the evolution of damage has a dominant effect on the micromagnetic parameters because the number of voids in the initial condition is very large and the surfaces were prepared to make negligible the effect of roughness and residual stresses. In constant- and multiple-amplitude (load increase) tests, the influence of damage on the fatigue behavior was investigated successfully. A significant difference between the fatigue behavior in the low-cycle fatigue and high-cycle fatigue ranges was found. The hot flat rolling process significantly reduced the number of voids while increasing the fatigue strength.

In the research undertaken by Gao, Z. J. et al. [17], the microstructure and the strain partitioning of lean duplex stainless steel 2101 (LDX 2101) during different hot-rolling processes are investigated by optical microscopy and electron-backscattered diffraction (EBSD). The results show that the LDX 2101 exhibits poor thermoplasticity at high temperatures. The four-pass hot-rolled plates show fewer edge-cracking defects and superior thermoplasticity compared with the two-pass hot-rolled plates prepared at different temperatures.

A trio of authors, Zhang, H. L., Feng, G. H. and Cui, H. Z. [18], carried out research about the analysis of crack defects in Q420B angle steel during the hot-rolling process. They found that in order to solve the problem of increased folding and scratching in the hot-rolling process of Q420B large-size angle steel, the numerical simulation method can be used to study the deformation characteristics of the W butterfly type. By modifying the hole profile of the fifth pass of the W butterfly type, the surface quality problems in the production process of large-size angle steel were significantly improved. The results show that a larger size of angle steel can be rolled out with a smaller billet by the W-pass system, but the angle steel needs to be bent several times for the W-pass system.

The team led by Cometa, A. [19] deals with the problems that may occur during the rolling of metal sheets defects, such as local waviness, surface ruptures, and—sometimes—strip breaks. These phenomena, commonly referred to as “pinching”, have been observed in combination with snaking problems (strip sideward movements) during tailing, and even in continuous rolling processes. Severe pinches compromise the quality of the strip, and damage to the work rolls can also occur. Pinches occur due to disruptions in the rolling process, therefore pinching-sensitive operative regimes need to be identified such that mill operations can be performed in a way that keeps the process stable.

The authors Feng, X., Wang, X., Sun, J. and Yang, Q. [20] completed research on the analysis of the tapered work roll shifting technique in five-stand UCMW tandem cold-rolling process. The tapered work roll shifting technique is effective to reduce edge drop, but results in a rigid roll stack and strip breaks. The effects of bending and shifting of the work roll and intermediate roll on edge drop and profile were discussed. The roll stack becomes the most rigid at the second stand, which calls for extra intermediate roll shifting to prevent strip breaks. Both the control abilities of work roll bending and intermediate roll bending on edge drop and strip crown are greatly enlarged by the work roll shifting technique.

In the research undertaken by Ma, Q. L. et al. [21] is clear that cross breaks are formed on the surface of cold-rolled and tempered strips during temper rolling, influencing the surface quality and mechanical characteristics of the temper rolled strip. By analyzing the loading of strip at S rolls and the formation mechanism and influencing factors of cross breaks, it was concluded that the main cause of cross breaks is the yield point elongation and the high tensile stress at the entrance of temper rolling mill.

A trio of authors, Garber, E. A., Shalaevskii, D. L. and Traino, A. I. [22], completed research on the simulation of cold rolling and skin rolling of strips in the deformation zone consisting only of a forward slip zone. The results of the simulation and investigation of the conditions of rolling and skin rolling of steel strips demonstrate that the deformation zone, consisting only of a forward creep zone, can be created in the working stands of mills by increasing the forward tension forces. Therefore, such a deformation zone in a combined algorithm for the energy–force calculation of such mills should be considered along with other structure types of the deformation zone.

The research paper produced by the authors Kozhevnikov, A. V., Shalaevsky, D. L. and Smirnov, A. S. [23] considers an engineering approach applicable to the design of technology for rolling cold-rolled strips that eliminate the occurrence of negative vibration effects (the phenomenon of “chatter”). It is found and verified that the elimination of “chatter” in the rolling stand requires the preservation of the sign of the difference of the strip volume during rolling. It is necessary to consider the actual thickness difference along the length of the strip. An analytical dependence of the effect of the tension of the rolled strip on the value of second volumes of the metal, which can also be used in the design of the technology, is obtained.

In the paper produced by the team led by Zhao, Y. [24], the vision-based automatic detection techniques proposed for the cold-rolling process to detect steel surface defects covered by industrial liquids and monitor the cold-rolling system based on the qualification of the industrial liquid regions numbers and sizes are demonstrated. The proposed method can detect most cracks and scratches accurately. All the experiments achieve an accuracy of more than 91%, although there is a big interference induced by industrial liquids and surface textures during the detection process.

In a research paper from the team led by Bahrami, A. [25], cracks were rather evenly distributed over the surface in the form of colonies of cracks. Samples were cut from the heavy plate. The results show that cracks are heavily oxidized. De-carburized areas are also seen alongside the cracks. The crack tip is in the form of a deer horn, indicating that crack branching took place during deformation.

## 2. Materials and Methods

The cold-rolling process is a continuous thickness reduction process in which a hot-rolled pickled strip is rolled between a pair of rotating work rolls to a customer-ordered gauge. Rolling is carried out on tandem mills or reverse mills. The main task of the cold-rolling mill is to produce a quality product, with the most accurate gauge. On modern cold-rolling mills are common hydraulic stands with automatic gauge and shape control. Cold-rolling tandem mills usually have four, five or six rolling stands in tandem depending on the type of rolled assortment. In the case of high-strength steel rolling, there is a requirement for making reduction to ordered gauge on rolling mills with a higher number of stands. 

**Figure 1:**
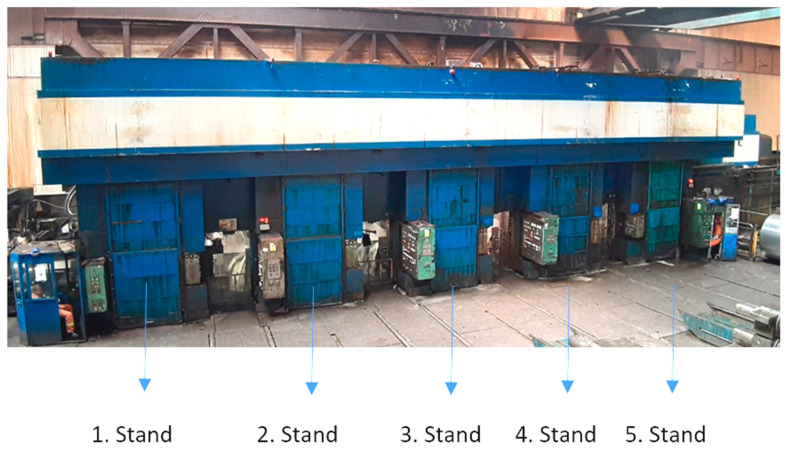
Five-stand cold-rolling tandem mill.

Figure 1 shows a five-stand cold-rolling tandem mill in which the presented research was carried out. Entry maximum thickness of strip is 3.8 mm exit thickness is maximum 1.75 mm, depending on the rolled steel grade. The research was carried out on tinplate steel grades, such as TH620N, TH415, TH550, TS290, TS245, and others. Tinplate grades are the most critical ones due to their very low thickness, around 0.175 mm, i.e., the thickness is comparable to a paper sheet.

**Figure 2:**
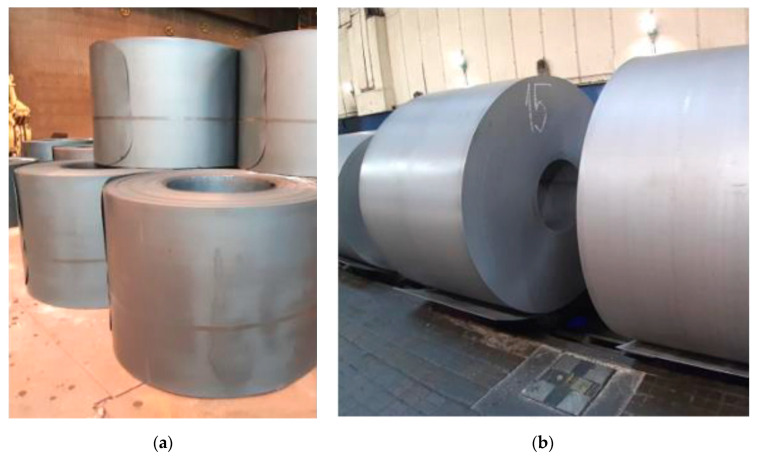
(**a**) Hot-rolled coils before pickling process; (**b**) Coils after pickling process.

The cold-rolling process is preceded by pickling. The pickling process is a process when oxides and other foreign materials are removed from the surface of a steel strip using acid solution. Figure 2a shows that the hot-rolled steel strip surface is dark, caused by oxides, and sometimes there is corrosion. Additionally, grease can occur. The cleaning of the strip surface is necessary for further production operations. Figure 2b shows coils with pickled surfaces.

The research was performed to determine the influence of the foreign particles on the strip-breaking mechanism in the rolling process and to reduce the number of strip breaks in cold-rolling mills. Empirical experience, based on historical database, was used in the research. 

The pivotal idea of the research was the identification of the critical defects in processes that precede rolling and subsequent taking of a corrective action in order to eliminate strip breaks. From the perspective of maximizing the success rate of detection and classification of critical defects, the most suitable process is steel strip pickling. In the pickling process, the scale layer and other foreign particles are removed from the strip surface—this allows for detailed inspection of the steel strip surface and detection of critical defects. Powerful camera systems are used to inspect the strip surface in the pickling process—these are capable of detecting and classifying defects. The detection of critical defects allows one to take preventive/corrective actions to reduce the risk of strip breaks.

The second step, after the detection and identification of critical defects on the strip during pickling, was a proposal of actions to reduce the risk of strip breaks.

The process of proposing the ideal correction was based on empirical experience and the theory of kinetic energy reduction in the rolling system. The kinetic energy reduction was expected to reduce the effect of the forces in the area of the critical defect that causes the destruction of the strip. This theory was based on the reduction of the rolling speed at the place where the defect, evaluated as “strip-break critical” during the pickling process, was present.

**Figure 3:**
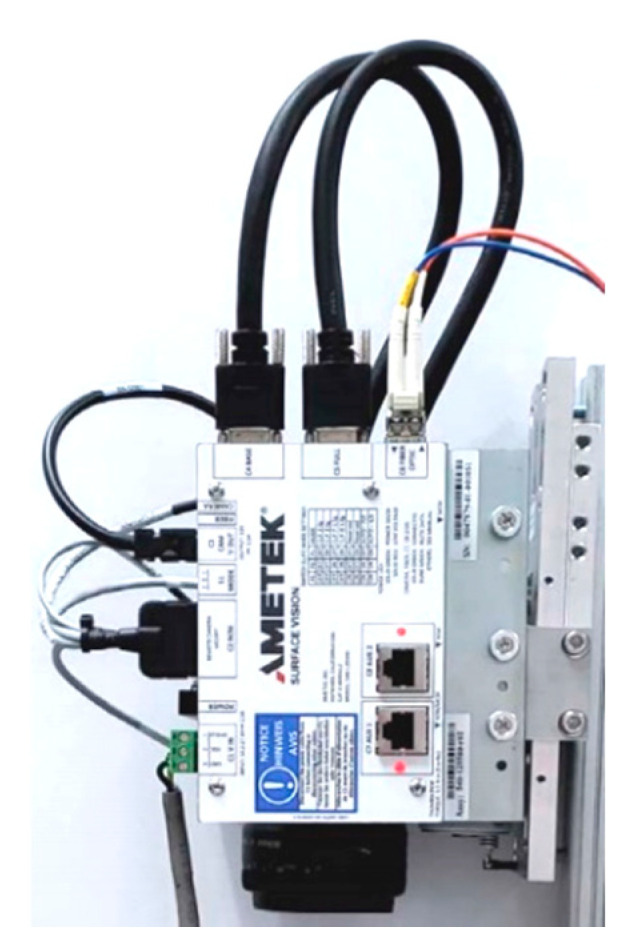
Surface inspection camera.

Figure 3 shows the camera with Camera Link Interface module (AMETEK Surface Vision, Hayward, WI, USA) which is installed at the pickling line. In total, eight cameras were installed at the pickling line: four on the top side and four on the bottom side. 

The SmartView system [26] uses linescan cameras to continuously inspect a moving web and detect defects in the material. These cameras contain a group of light sensors (picture elements or pixels) that build up electrical charge proportional to the light that reaches them (called exposure). The pixels are then digitized and shifted out of the camera. This process is repeated thousands of times per second. The rate at which this is performed is called the camera scan rate and is measured in linescans per second.

The camera works with 320 MHz speed with 6k resolution, providing a 53.3 kHz line rate. However, there is limitation on the SPU side, allowing us to process only 320 Mega pixels (understood as MHz) per second, so the speed of the camera is reduced to a maximum 160 MHz (26.6 kHz line rate) to serve two cameras.

**Figure 4:**
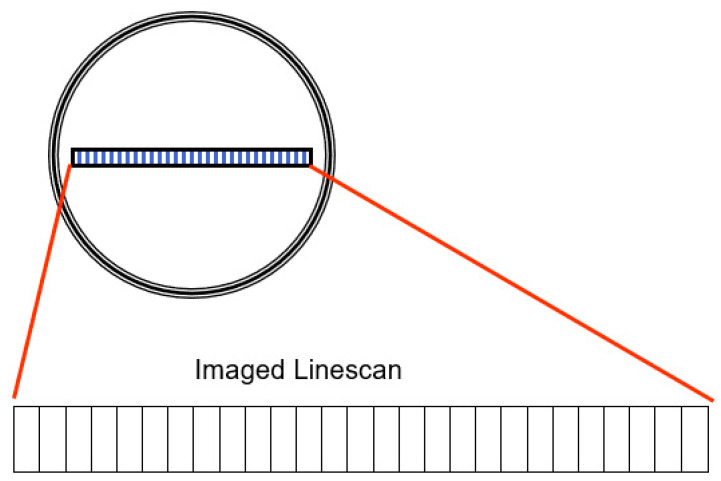
Array of SmartView camera, with single row of pixels.

Some cameras, such as those used in modern digital cameras, have a group of pixels arranged in rows and columns forming a rectangular shape (like the rectangular film of a standard photographic camera). Figure 4 shows SmartView cameras, however, there is a single row of pixels, and the cameras are therefore called linescan cameras because they see (scan) one line at a time.

**Figure 5:**
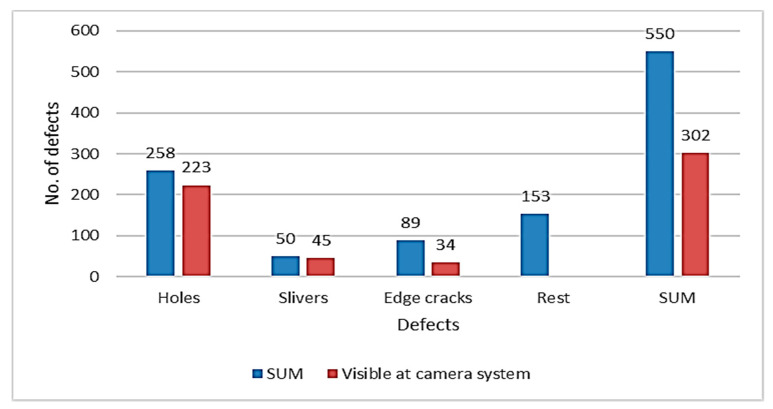
Result of defect verification.

The classification helps determine what defects are critical in the rolling process. System is able to identify defects that are present on strip surface and to predict the risk degree they pose to the rolling process. Figure 5 shows correlation of broken strips and defects visible on strip during pickling was made. There were 550 defects in the database; of that, 302 defects were visible on camera system records. The analysis shows, that preventive actions could have been implemented to prevent an incident during cold-rolling process. The data are displayed in Figure 5. The defects visible on camera records were present on 54.9% of defective coils.

**Figure 6:**
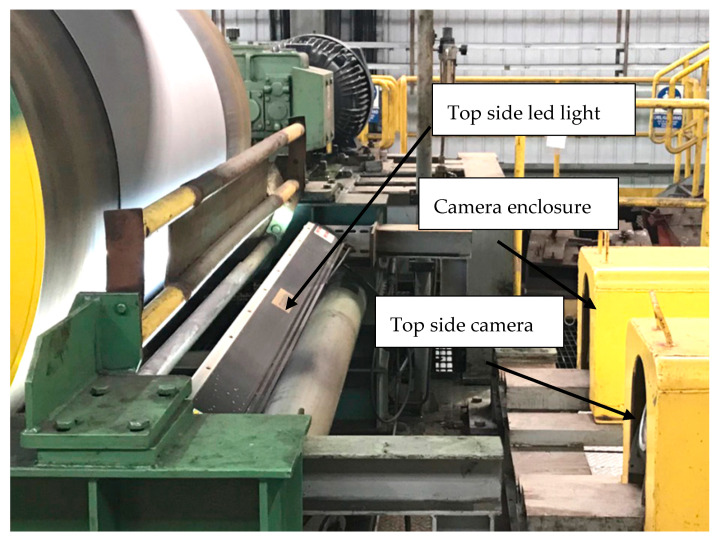
The SmartView system at the end of pickling line.

High-speed cameras were installed at Pickling Line Ametek SmartView®-Surface Inspection system (Hayward, WI, USA) with 6 K cameras; Ametek SmartView® scans the surface of the strip and categorizes the defects. The cameras are mounted in a protective enclosure against damage Figure 6. The surface of the strip lit by an LED light. 

**Figure 7:**
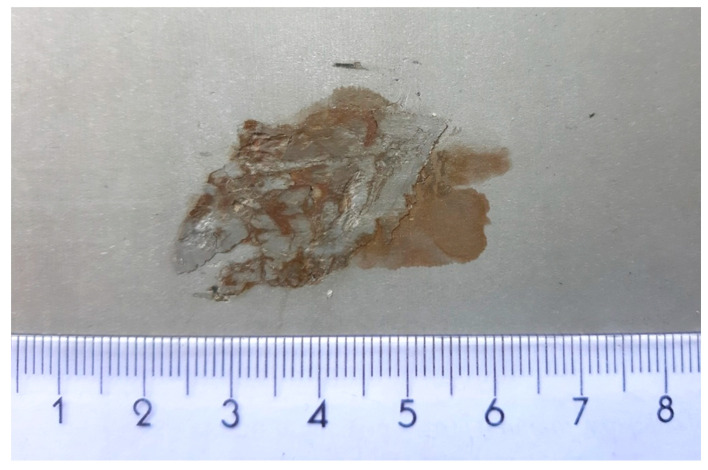
Foreign material inside strip (cm).

Figure 7 shows hot-rolled foreign material rolled on the surface in the hot-rolling process. Those pieces of material from hot-rolling mill fell to the strip surface and were rolled in. This comes from part of the hot-rolling mill and it is critical for the cold-rolling process. The foreign material was detected on the pickling line camera system and the sample was taken for verification.

**Figure 8:**
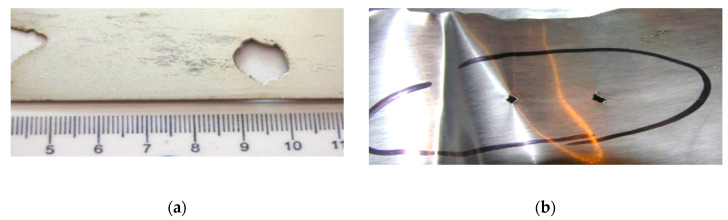
Samples from metallographic analysis (**a**) Holes in the strip after metallurgical sliver falls out after the rolling process (cm); (**b**) Holes in the strip after metallurgical sliver falls out after the rolling process.

**Figure 9:**
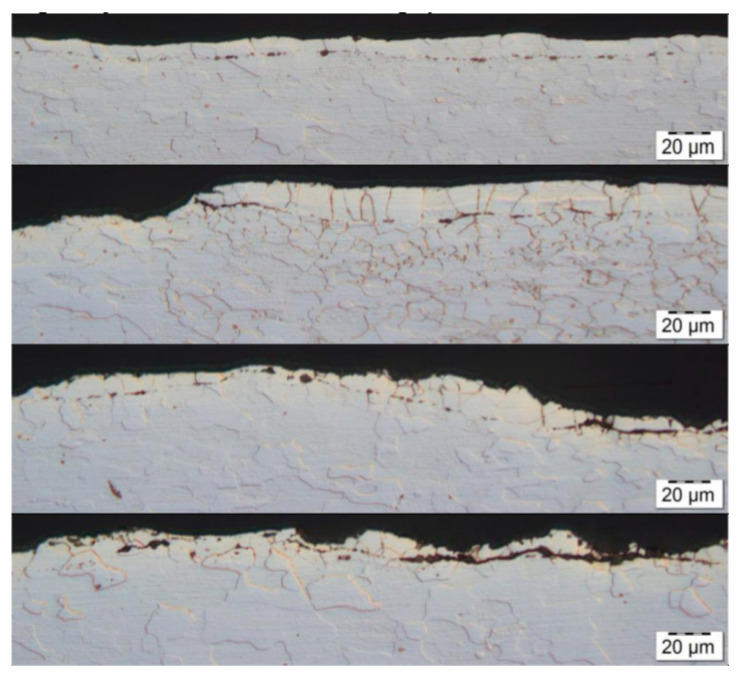
Optical micrographs of metallographic cross-section in the selected defective area.

**Figure 10:**
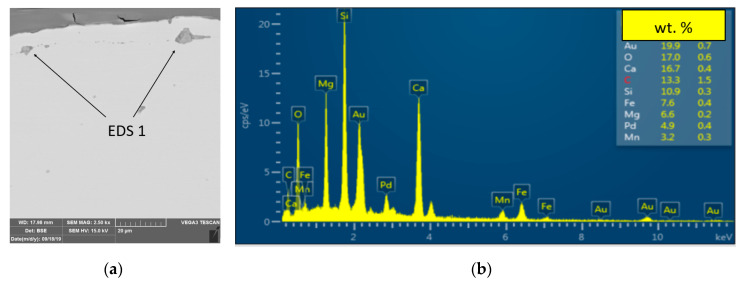
EDS, Energy-dispersive X-ray spectroscopy analyses of foreign fragments of samples: (**a**) Foreign particles in the steel substrate in the damaged area where the EDS analysis was performed (**b**) Results of EDS analysis, chemical elements detected by EDS analysis.

Further verification was provided after the cold-rolling process. The metallographic analysis confirmed that the root cause of the defect was a metallurgical sliver Figure 8. In the subsurface layers of the steel substrate were linear particles of steelmaking mold flux/cover flux Figure 9. The following elements were found in the particles: Si-Ca-Mg-O, Figure 10, EDS1.

## 3. Results

To verify the theory, a trial was made, and a telegram was implemented into the data flow between the Pickling line and Cold-Rolling Tandem at Level 2. The telegram transferred the information regarding the position of the simulated defect on the strip, as well as the place where it was necessary to reduce the rolling speed. After rolling the simulated critical defect, the rolling mill increased the rolling speed to the original value.

A total of 100 coils was selected during the research. These were subjected to the simulated rolling of the critical defect, i.e., rolling speed correction was carried out during their processing. After the test, the forces in the rolling mill were evaluated. The following parameters were reviewed: total roll forces, differential tensions, inter-stand tensions and the last stand tilting, as it significantly influences the flatness of the rolled strip.

**Figure 11:**
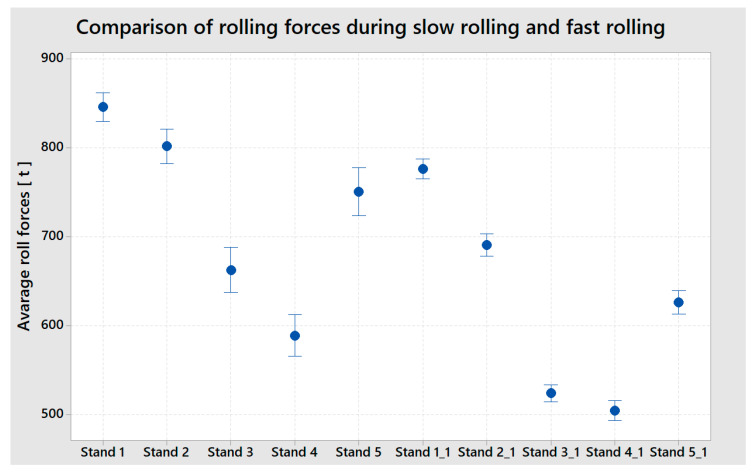
Comparison of rolling forces slow rolling (Stand 1, 2, 3, 4, 5,) and fast rolling (Stand 1_1, 2_1, 3_1, 4_1, 5_1).

**Figure 12:**
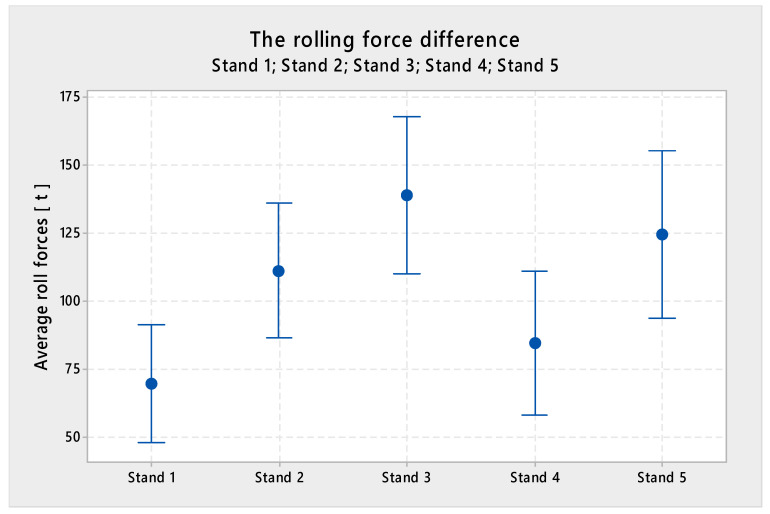
Difference of forces slow and fast rolling.

The results did not confirm our expectations. Based on the empirical experience, it was expected that through kinetic energy reduction, the forces in the rolling process would also drop, thus reducing the risk of a strip break. The results achieved during the trial are displayed in Figure 11 and Figure 12. The rolling speed reduction did not reduce the forces applied on the critical position in the strip where the simulated defect was located; on the contrary, the values measured in the rolling process were, when the rolling speed was reduced, higher than when the strip was rolled at a higher, stable rolling speed. The inter-stand tensions increased and so did the differential tensions, overall rolling forces, and the Stand 5 tilting was oscillating, and the thickness chart was much more disturbed than at the portion of the strip where the rolling speed was stable. The boxplot chart in Figure 11 shows the rolling force values in tons at each rolling stand at the time when the rolling speed was reduced to 900 m/min (Stand 1, 2, 3, 4, 5) and values in tons at each rolling stand during the stable rolling speed of 1250 m/min. (Stand 1_1, 2_1, 3_1, 4_1, 5_1)

The difference between the two rolling modes is clearly visible. When rolling at the stable speed of 1250 m/min, the rolling forces have a smaller scatter and the rolling process is more stable than when the rolling process slows down. The most significant finding is that the value of the rolling forces, i.e., the factor that most significantly influences the factors that negatively influence the rolled strip from the perspective of the potential destruction of a strip with a critical defect in the rolling process. The overall rolling forces at each stand are significantly higher at a lower rolling speed.

**Figure 13:**
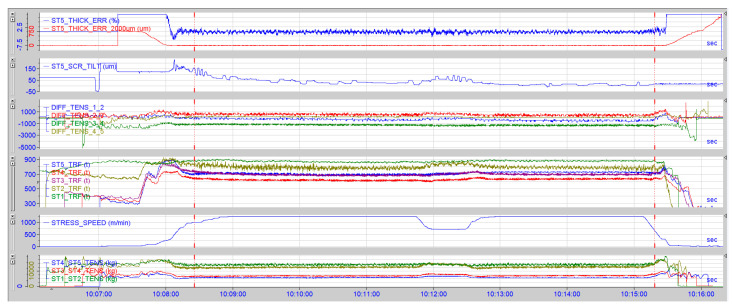
Forces during slow-down of the rolling process.

Figure 13 shows the difference between the average value of total rolling force at each stand at the reduced speed, created with a sample of 100 coils.

The conclusion:

(1) At Stand 1, the value was higher by 69.57 tons
(2) At Stand 2, the value was higher by 111.21 tons
(3) At Stand 3, the value was higher by 138.71 tons
(4) At Stand 4, the value was higher by 84.68 tons
(5) At Stand 5, the value was higher by 124.59 tons

In Figure 13, one can observe the forces captured by the Iba analyzer during the rolling process when the rolling was reduced; the ST5_THICK_ERR value displays the discrepancy from the requested strip thickness. It is clearly visible that the thickness was oscillating when the speed was reduced. Even though it is within the required limits, gauge oscillation is an unwanted phenomenon. The ST5_SCR_TILT chart displays the values from Stand 5 tilting where the stand was, due to the rolling parameters’ change induced by the slow-down, tilting +70 µm. The tilting of the last stand regulates the strip flatness; any unwanted changes of tilting may have a negative impact on the strip flatness. The following charts: DIFF_TENS (different tensions), TRF (total roll force), STX_STY_TENS (tension between stands) display oscillating forces in the rolling process incurred by the rolling speed change.

**Figure 14:**
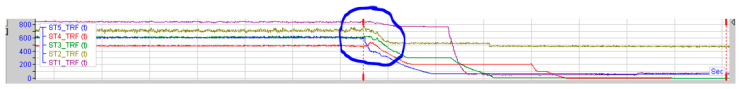
Rolling forces during a strip break.

Figure 14 shows the course of strip rolling and the moment when the strip broke during rolling. By looking at the trend of the forces, it is evident that at the moment of the destruction, as well as at the time just before the incident, there were no extraordinary deviations in the rolling process. Despite stable forces, the strip was destroyed due to the occurrence of a foreign material in the structure of the strip. Had the rolling speed been slowed down to prevent the strip break, this action would have had the very opposite effect—the forces would start oscillating and the risk of the strip break would have increased.

## 4. Discussion

Based on the research, we conclude that the rolling speed reduction does not eliminate strip breaks in the rolling process. Rolling speed reduction causes an increase of rolling forces, as well as energy in the rolling system, which negatively influences rolling. The reduction of the speed would result in an opposite effect and the risk of a strip break would increase. 

To eliminate strip breaks in rolling processes caused by foreign objects, it is necessary to take corrective actions in the process of their origination—steel slab casting. The effectivity of measures implemented in the downstream process steps is low.

The foreign material present on the strip surface would, in the rolling process, enter the inner structures and disrupt the material in its whole cross-section. This has a negative impact on the homogeneity of the steel strip. If the strip does not break, the foreign particles remain in the strip. If the foreign particles fall out of the strip during the rolling process, holes are left in the strip.

Such holes are a potential risk in the production processes that follow rolling, including strip breaks and product quality deterioration.

The subsequent research, aimed at the reduction of the risk of strip breaks during rolling, will focus on final strip thickness. If the final strip thickness is increased, the foreign particle would not penetrate the material structures deep enough to interfere with the whole cross section. It is believed that this should reduce the number of strip breaks. 

The final thickness change can only be completed once a foreign object is identified on the strip surface. The most suitable place for this operation is the exit of the pickling process—the cameras located in this area can identify critical defects on the strip surface.

## 5. Conclusions

Increasing the final thickness of the rolled strip and elimination of the surface defects at the place of their origin is the most cost-effective method of reducing their unwanted effect on the product quality and the risk of strip-breaks in the rolling process. A total (100%) elimination of defects is hardly achievable. The research of minimizing the influence of foreign particles on strip-breaks during rolling should focus on determining the characteristic properties of the critical rolled-in particles on the strip surface. To ensure their accurate determination, a database of the foreign particles and their properties needs to be developed. The properties can be determined by a camera system during pickling. Amongst the most critical defect properties are their dimensions, type and position on strip. Other important factors are the properties of the strip on which the defect is present, such as the strip gauge at the entry of the rolling mill, its width, the exit gauge, the reduction, the rolling speed, the draft plan and the steel grade.

Once the database containing the above-mentioned parameters is developed, the rolling process and its results (strip breaks) need to be evaluated and the correlation between the defects’ parameters and strip breaks must be verified.

Presently, there is no effective predictive calculation or mathematic model for the determination of the properties of the defects that cause a strip break. Detecting the parameters that would accurately predict strip-breaks should be possible using an experimental database. After developing the database, all of the findings could be summarized and a “strip-break during rolling process prediction theory” could be developed.
